# Application of multi-criteria decision analysis techniques and decision support framework for informing arbovirus risk assessments for planning, preparedness and response

**DOI:** 10.3389/fbioe.2025.1682355

**Published:** 2025-12-03

**Authors:** Segaran P. Pillai, Elizabeth Fox, Ann M. Powers, Stephen A. Morse

**Affiliations:** 1 U.S. Department of Health and Human Services, Food and Drug Administration, Office of the Commissioner, Silver Spring, MD, United States; 2 Centers for Disease Control and Prevention, Fort Collins, CO, United States; 3 Centers for Disease Control and Prevention (Retired), Atlanta, GA, United States

**Keywords:** arbovirus, risk assessment, multi criteria decision analysis, decision support framework, planning, preparedness and response

## Abstract

**Introduction:**

Globally, more than 17% of human infections are caused by vector-borne viruses, which result in more than 700,000 deaths annually as per the World Health Organization. Mosquitoes and ticks are the primary arthropod vectors, along with sandflies and midges. More than 500 arthropod-borne viruses (arboviruses) have been described, with more than 150 causing human disease. It is important to understand the public health risk associated with arboviruses.

**Methods:**

We used multi-criteria decision analysis (MCDA) techniques and a Decision Support Framework (DSF) employing a logic tree format to identify high-risk arboviruses, applying these approaches to only those arboviruses transmitted by flying insects (i.e., mosquitos, sandflies, and midges) due to their potential for efficient transmission and habitat expansion.

**Results:**

A literature review of 54 arboviruses against 13 criteria was conducted for assessing risk and documenting the findings that support this assessment. The most prominent data gaps found were those for the annual global incidence, the severity of disease, and long-term impact. Technical review of published data and associated scoring recommendations by subject matter experts (SMEs) were found to be critical, particularly for pathogens with very few known cases. The MCDA analysis supported the intuitive sense that agents with high mortality and morbidity rates should rank higher on the relative risk scale when considering disease persistence and severity. However, comparing scores to suggest thresholds for designating high risk versus (vs) moderate risk vs low risk, was challenging and will require additional real time data during an outbreak. The DSF utilized a logic tree approach to identify arboviruses that were of sufficiently low enough concern that they could be ruled out from further consideration. In contrast to the MCDA approach, the DSF ruled out an arbovirus if it failed to meet even one criteria threshold.

**Conclusion:**

The MCDA and DSF approaches arrived at similar conclusions, suggesting that using these analytical approaches for an arbovirus risk assessment added robustness for decision making.

## Introduction

Arboviruses comprise a heterogeneous group of viruses, which is defined by the epidemiological fact that they are transmitted between vertebrate hosts via biting and blood-sucking arthropods (Artsob et al., 2023). The primary arthropod vectors are mosquitoes and ticks, along with sandflies and midges (Artsob et al., 2023). More than 150 arboviruses are known to cause human disease (Madewell, 2020). Many of these viruses are considered emerging pathogens based on their geographic spread and their increasing impact on susceptible human populations (LaBeaud et al., 2011). In their acute stages, arboviral infections cause a broad spectrum of disease, ranging from asymptomatic infections to severe undifferentiated fever (CDC, 2024a). Arboviral infections can also progress to much more complex secondary conditions, or sequelae, such as encephalitis or hemorrhage, which can result in long-term physical and cognitive impairment or in early death (CDC, 2024a; Hollidge et al., 2010). However, even the milder forms of arbovirus infection can result in long-lasting impairment (Carson et al., 2006; Sejvar et al., 2007). Arboviruses are found in diverse viral families: (i) Peribunyaviridae (genus *Orthobunyavirus*), Nairoviridae (genus *Orthonairovirus)*, and Phenuiviridae (genera *Phlebovirus*, *Bandavirus*, and *Uukuvirus*), (ii) Flaviviridae (genus *Orthoflavivirus*), (iii) Sedoreoviridae (genus *Orbivirus*), (iv) Rhabdoviridae (genus *Vesiculovirus*), (v) Togaviridae (genus *Alphavirus*), (vi) Spinareoviridae (genus *Coltivirus*), and (vii) Asfarviridae (genus *Asfarvirus*) (Weaver and Reisen, 2010; ICTV, 2025). Most arboviruses are RNA viruses. Peribunyaviridae, Nairoviridae, Phenuiviridae, and Rhabdoviridae are negative-sense single-stranded RNA viruses; Flaviviridae and Togaviridae are positive-sense single-stranded RNA viruses; and Sedoreoviridae and Spinareoviridae are double stranded RNA viruses. The only known DNA arbovirus belongs to the Asfarviridae family (Van Regenmortel et al., 2000).

All arborvirus disease cycles (i.e., episystems) are comprised of dynamic interactions between the arthropod vector, the specific virus, and the vertebrate host, which sometimes includes amplification and dead-end hosts (Tabachnick et al., 2014). These interactions are complex and can be influenced by diverse factors such as poverty, environmental and cultural conditions, land and water use practices, human behavior, human and animal population size and growth, and human travel and commerce (Tabachnick et al., 2014). Furthermore, many of the environmental factors may be directly or indirectly influenced by weather and climate. The resulting variables may have a positive, negative, or neutral effect on disease transmission (Tabachnick et al., 2014).

Several important human pathogens are arboviruses. For example, Yellow Fever virus (YFV, Family Flaviviridae, Genus *Orthoflavivirus*) originated in Africa and together with its vector spread to the New World with the slave trade in the mid-17th century (Bryant et al., 2007). It is estimated that there are 30,000–200,000 clinical cases of yellow fever per year (Mutebi and Barrett, 2002). The disease caused by YFV is associated with high mortality rates; 15%–25% of early symptomatic cases progress to a more severe hemorrhagic form, which has a mortality rate of 20%–50% (Gubler, 2004). YFV is primarily transmitted between humans by *Aedes aegypti* as a domestic/peridomestic disease (Monath, 2001).

Dengue virus (DENV, Family Flaviviridae, Genus *Orthoflavivirus*) first appeared in the New World about the same time as yellow fever suggesting that DENV and YFV were imported on the same slave ships together with the historically African mosquito *A*. *aegypti* (Braak et al., 2018). Dengue has spread to more than 120 countries mostly in the tropics and subtropics (Braak et al., 2018). DENV is a complex of four phylogenetically and antigenically distinct serotypes causing fever (DF) with or without hemorrhage (DHF), shock, or death in humans. It is estimated that there are 50–100 million cases of dengue fever and 250,000 - 500,000 cases of DHF each year throughout the world. The case fatality rate in adults with these more severe forms ranges from <1% to 20% (Medagama et al., 2020). DENV is transmitted among humans in urban environments primarily by *A. aegypti* and by *Aedes albopictus* in peri urban and rural environments (Braak et al., 2018).

West Nile virus (WNV, Family Flaviviridae, Genus *Orthoflavivirus*) was first isolated in 1937 from the blood of a patient in Uganda (Smithburn et al., 1940). Phylogenetic trees constructed from many isolates suggest that WNV originated in Africa and spread via migratory birds throughout Africa, the Middle East, Europe, India, the Americas, and Australia and is now considered the most important cause of viral encephalitis worldwide (Chancey et al., 2015). WNV was introduced into New York in 1999 and has subsequently spread across the U.S, Canada, Mexico, and the Caribbean (Gubler, 2007). Between 1999 and 2013, WNV caused 17,463 cases of neuroinvasive disease and 1668 fatalities in the U.S. However, most human WNV infections are asymptomatic but often manifest as febrile illness with headache, rash, fatigue, myalgia, and arthralgia; severe infections may lead to paralysis, seizures, or cerebellar ataxia with associated long-term cognitive and neurological impairment (Petersen et al., 2013). The mortality rate is close to 10% among patients with neuroinvasive disease (Hollidge et al., 2010). Human fatalities are mostly associated with young children and elderly patients (Petersen et al., 2013). Other vertebrates such as horses are also susceptible to WNV (Gubler, 2007). WNV uses a wide range of bird species as amplifying hosts and is transmitted by various species of *Culex* mosquitoes including members of the *C. pipiens* complex. The North American members of this complex are *Culex pipiens pipiens* and *C. pipiens quinquefaciatus* (Andreadis, 2012; Tabachnick et al., 2014). *Culex tarsalis* has emerged as the primary vector in the Western U. S. and *Culex nigripalpus* is an important vector in the Southeastern U. S. (Tabachnick et al., 2014). Neither species had ever been previously exposed to WNV, but once the virus was introduced into their geographic ranges, both species proved to be highly competent WNV vectors (Tabachnick et al., 2014). Other important arboviruses that belong to the family Flaviviridae are Zika virus (ZIKAV), Japanese encephalitis virus (JEV), and St Louis encephalitis virus (SLEV) (Weaver and Barrett, 2004).

Chikungunya is an infection caused by the Chikungunya virus (CHIKV, Family Togaviridae, Genus *Alphavirus*). The disease was first identified in Tanganyika (now Tanzania) in 1952 (Robinson, 1955). Its name is based on the Kimakonde words “to become contorted,” which likely refers to the contorted posture of people affected with the severe joint pain and arthritic symptoms associated with the infection (Caglioti et al., 2013). Other symptoms may include headache, muscle pain, swollen joints, and a rash (Caglioti et al., 2013). Newborns infected around the time of birth, older adults (≥65 years), and those with medical conditions (e.g., high blood pressure, diabetes, heart disease) are at risk for more severe disease (CDC, 2024b). The fatality rate is estimated to be ca. 1/1,000 (Caglioti et al., 2013). The virus is spread by *A. aegypti* and *A. albopictus* (Caglioti et al., 2013). Cases and outbreaks have been identified in >100 countries in the Americas, Africa, Asia, Europe, and the Indian and Pacific Oceans (CDC, 2024b). CHIKV is the epidemiologically most prevalent alphavirus transmitted to humans by *Aedes* mosquitoes during their blood meal (Kril et al., 2021). Phylogenetic analysis has identified three distinct lineages of CHIKV corresponding to their respective geographical origins: the West African, the East-Central-South African, and the Asian lineages (Brault et al., 2000; Schuffenecker et al., 2006). Before 2006, CHIKV disease was rarely identified in the U.S.; between 2006–2013, there was an average of 28 cases per year of travel-associated CHIKV infections. In 2014 and 2015, locally acquired cases were reported from Florida (N = 14) and Texas (N = 1), meaning that mosquitoes in the area had been infected with CHIKV and were spreading it to people (CDC, 2024b). Venezuelan Equine Encephalitis virus (VEEV), Western Equine Encephalitis virus (WEEV), and Eastern Equine Encephalitis virus (EEEV) are additional alphaviruses of importance.

The role of vectors and their feeding preferences (anthropophilic and/or ornithophilic) are of major importance in global or local spread of arboviral infections (Kuno and Chang, 2005). Five human epidemic mosquito-borne arboviruses, YFV, DENV, WNV, CHIKV, and ZIKAV, have emerged in both hemispheres in recent centuries. Other mosquito-borne arborviruses (e.g., JEV, Murray Valley encephalitis virus (MVEV), Rift Valley Fever virus (RVFV), Usutu virus (USUV), Spondweni virus (SPOV), and O’nyong nyong virus (ONNV) have emerged in specific regions of the world but are not yet found in both hemispheres.

Nonbiological transmission (i.e., direct and mechanical) of many arboviruses can also occur. Direct transmission can occur via intranasal, oral, venereal, exposure of skin with abrasions, cornea, reproductive tissue, or any mucous membrane (Kuno and Chang, 2005). Because of the risk of aerosol exposure when working with arboviral cultures, the BMBL has recommended the following biosafety levels (see Table 1 for abbreviations): JEV, CHIKV, EEEV, VEEV, WEEV, YFV, RVFV, WNV, SLEV, WESV, MUCV, NRIV, SFV, GERV, BAV and MVEV are BSL-3, while BAGV, BANV, ZIKAV, NTAV, UGSV, MAYV, ONNV, SINV, RRV, ORUV, KAMV, MOSV, VSVAV, CHPV, LACV, CEV, JCV, TAHV, BUNV, BWAV, ILEV, SFNV, NDV, PGAV, SFSV, SHUV, TCMV, TOSV, WITV, OROV, BFV, LUMV, TUCV, MIDV, NDUV, VSIV, VSNJV, SPOV, USUV, and DENV-1-4 are BSL-2 (CDC/NIH, 2020).

**TABLE 1:** List of arboviruses subjected to risk assessment.

Flaviviridae (14)		Togaviridae (13)		Sedoreoviridae (2)		Rhabdoviridae (4)		Peribunyaviridae, Nairoviridae and Phenuiviridae (21)	
Yellow Fever virus	YFV	Chikungunya virus	CHIKV	Orungo virus	ORUV	Kamese virus	KAMV	Rift Valley Fever Virus	RVFV
Dengue virus	DENV	Western Equine encephalitis virus	WEEV	Banna virus	BAV	Mossuril virus	MOSV	La Crosse encephalitis virus	LACV
Zika virus	ZIKAV	Eastern Equine encephalitis virus	EEEV			Vesicular stomatitis virus (Alagoas, Indiana, Jersey)	VSV VSAV VSIV VSNJV	California encephalitis virus	CEV
Japanese encephalitis virus	JEV	Venezuelan Equine encephalitis virus	VEEV			Chandipura virus	CHPV	Jamestown Canyon virus	JCV
West Nile virus	WNV	Barmah Forest virus	BFV					Tahyna virus	TAHV
Bagaza virus	BAGV	Middelburg virus	MIDV					Bunyamwera virus	BUNV
St. Louis encephalitis virus	SLEV	Mayaro virus	MAYV					Bwamba virus	BWAV
Banzi virus	BANV	Mucambo virus	MUCV					Germiston virus	GERV
Ntaya virus	NTAV	Ndumu virus	NDUV					Ilesha virus	ILEV
Murray Valley encephalitis virus	MVEV	O’Nyong-Nyong virus	ONNV					Lumbo virus	LUMV
Spondweni virus	SPOV	Semliki Forest virus	SFV					Sandfly Fever Naples virus	SFNV
Uganda S virus	UGSV	Sindbis virus	SINV					Ngari virus	NRIV
Usutu virus	USUV	Ross River virus	RRV					Nyando virus	NDOV
Wesselsbron virus	WESV							Oropouche virus	OROV
								Pongola virus	PGAV
								Sandfly Fever Sicilian virus	SFSV
								Shuni virus	SHUV
								Tacaiuma virus	TCMV
								Tucunduba virus	TUCV
								Toscana virus	TOSV
								Witwatersrand virus	WITV

Arboviruses have been considered for use as biological weapons and some have even been weaponized by state biological weapons programs (WHO, 1970; James Martin Center for Nonproliferation Studies, 2024). Using YFV as an example, case fatality rates up to 30%–40% are not uncommon in unvaccinated individuals living in urban or rural areas when YF is newly introduced. YFV can be grown in large amounts in eggs or tissue culture and freeze dried. Aerosol transmission of YF has been achieved in laboratory studies. In another example, the Far Eastern subtype of tick-borne encephalitis virus (TBEV) can be easily grown *in vitro*, and its high infectivity and lethality by the aerosol route could result in a case fatality rate of 25% (WHO, 1970). JEV can also be easily propagated *in vitro* and populations outside endemic areas are universally susceptible. Since aerosol infection of animals has been achieved in laboratory studies, it is reasonable to assume that JEV can be disseminated by aerosols (WHO, 1970). DENV, VEEV, CHIKV, ONNV, and RVFV can all be propagated *in vitro*. Susceptible populations and aerosol delivery also make these viruses potential biological weapons. In 1970, a WHO Expert Committee estimated that the release of 50 kg of RVFV or TBEV along a 2 km line upwind of a population center of 500,000 would result in 400 dead and 35,000 individuals incapacitated and 9,500 dead and 35,000 incapacitated, respectively (WHO, 1970). For comparison, they estimated that a similar release of anthrax spores would result in an estimated 95,000 dead and 125,000 incapacitated. The differences can be attributed to a 300-fold difference in the decay rates of the virus vs. spores (WHO, 1970). In another example, VEEV was developed and stockpiled as an incapacitating agent by the U.S. biological weapons program; it was later destroyed in 1971–1973 (Christopher et al., 1999). VEEV was also weaponized by the USSR bioweapons program (Alibek and Handelman, 1999). Several countries have conducted research on various arboviruses as potential biological weapons: Canada, YFV; North Korea, YFV; USSR, JEV, Russian Spring-Summer Encephalitis Virus (RSSEV), YFV, and ASFV; and the U.S, EEEV, WEEV, YFV, DENV, RVFV, and CHIKV (James Martin Center for Nonproliferation Studies).

We conducted a risk assessment using MCDA techniques and a DSF to better understand the risk that arboviruses pose to human health. MCDA is a sub-discipline of operations research that evaluates multiple conflicting criteria in decision making. It is comprised of a set of methodological approaches that are well documented in the literature for conducting structured risk assessments (Keeney and Raiffa, 1993; Hwang and Kawngsun, 2012; Greco et al., 2016; Linkov et al., 2006). The use of MCDA for risk-based decision making has been described for environmental applications (Kiker et al., 2005; Steele et al., 2009), healthcare (Velasquez and Haster, 2013; Thokala et al., 2016), as well as emerging threats to animal and plant health (Cook and Proctor, 2007) and foodborne pathogens (Ruzante et al., 2010). MCDA allows for data uncertainty, can combine multiple information sources including those based on expert judgement, and is simple in concept and amenable to a user-friendly software tool. Disadvantages of MCDA include those cited for qualitative measurements, i.e., the lack of absolute measurements, and the potential for rank reversal (Cox et al., 2005). We have previously used MCDA to inform select agent and toxin designation (Pillai S. P. et al., 2022; Pillai S. P. et al., 2022; Pillai et al., 2023a; Pillai et al., 2023b).

## Methods

### Analytical framework

**FIGURE 1:**
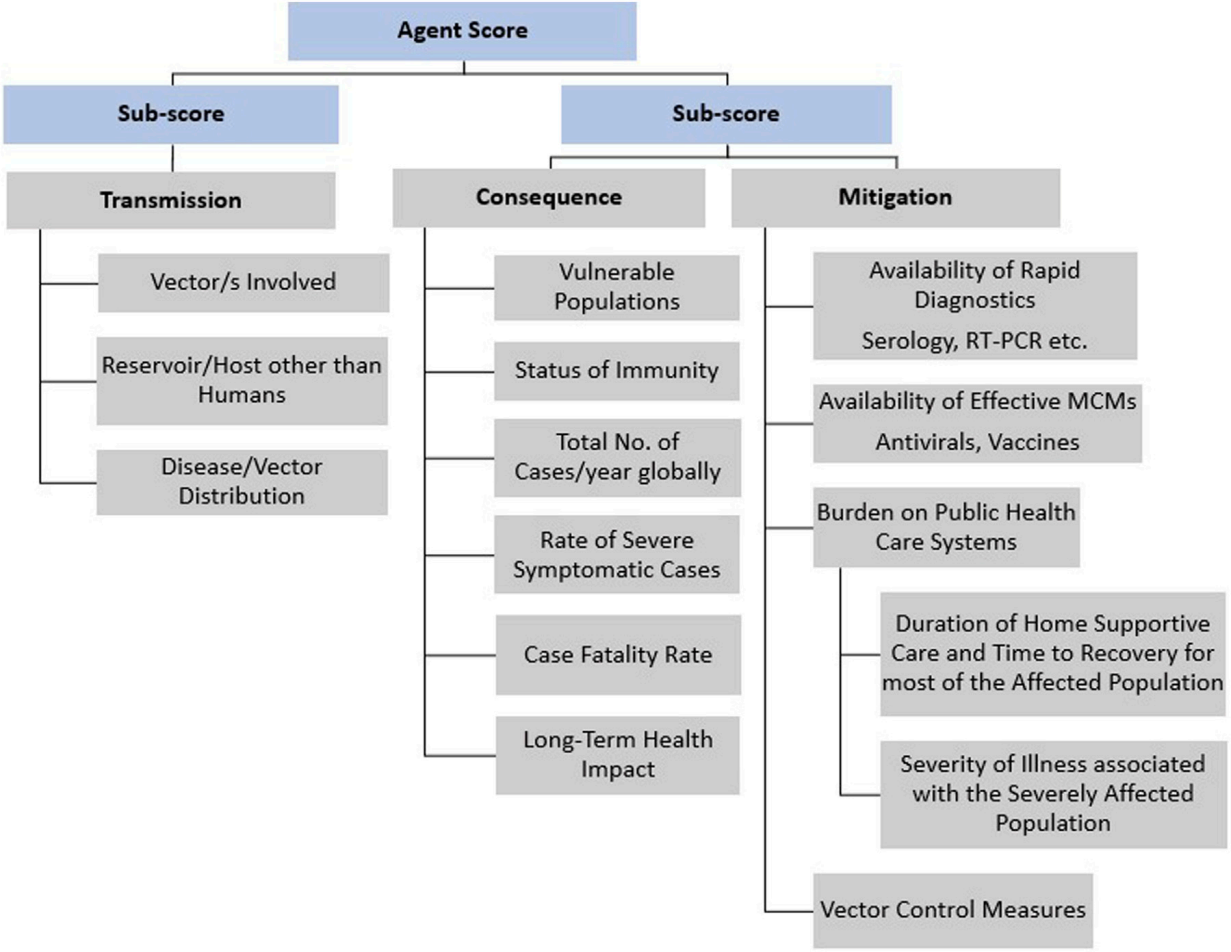
Summary of the criteria and hierarchy captured in the MCDA tool for Arbovirus Risk Assessment.

The starting point for the MCDA analysis was a set of 13 criteria (Figure 1) that supports arbovirus risk. These criteria were chosen based on public health impact and response, which encompasses the following: i.) disease transmission, (associated with vectors involved, distribution and persistence); ii.) consequence (associated with vulnerable population and disease impact); and iii.) mitigation (associated with our ability to respond). The results of an extensive literature search and SME input contributed to the scoring of these 13 criteria on a scale of 0–10 (see Tables 2–4), based on the scoring definitions in Table 5, for each of the arboviruses listed in Table 1. The scoring scale reflects relative concern as it pertains to the agent’s designation of risk concern, with 0 corresponding to no concern and 10 corresponding to highest concern. For simplicity, a linear scale was chosen for this evaluation. Table 5 lists the scoring definitions for each of the criteria for even-numbered scoring options: 0, 2, 4, 6, 8, and 10. In the event SMEs were not in agreement on an even-numbered score, which sometimes occurred for criteria with more qualitative data, we assigned odd-numbers as an intermediate score.

**TABLE 2:** “Transmission” sub-scores by agent.

Agent	Transmission sub-scores		
	Vector/s involved	Reservoir/Host other than human	Disease/Vector distribution
Yellow Fever virus	6	8	4
Dengue virus	5	4	10
Zika virus	6	4	10
Japanese encephalitis virus	10	9	4
West Nile virus	10	10	2
Bagaza virus	5	4	1
St. Louis encephalitis virus	10	6	2
Banzi virus	4	5	1
Ntaya virus	2	8	2
Murray Valley encephalitis virus	3	10	1
Spondweni virus	6	4	2
Uganda S virus	3	6	2
Usutu virus	10	10	4
Wesselsbron virus	9	7	2
Chikungunya virus	6	7	10
Western Equine encephalitis virus	6	10	1
Eastern Equine encephalitis virus	10	10	2
Venezuelan Equine encephalitis virus	10	6	2
Barmah Forest virus	6	8	1
Middelburg virus	6	7	1
Mayaro virus	6	6	1
Mucambo virus	7	6	1
Ndumu virus	6	6	1
O’Nyong-Nyong virus	3	4	1
Semliki Forest virus	8	8	2
Sindbis virus	10	9	4
Ross River virus	5	10	1
Orungo virus	7	8	1
Banna virus	4	5	1
Kamese virus	3	4	1
Mossuril virus	7	5	4
Vesicular stomatitis virus (Alagoas, Indiana, Jersey)	5	10	2
Chandipura virus	3	10	1
Rift Valley Fever Virus	5	8	4
La Crosse encephalitis virus	4	4	1
California encephalitis virus	8	2	1
Jamestown Canyon virus	9	4	1
Tahyna virus	10	6	2
Bunyamwera virus	5	7	2
Bwamba virus	3	5	1
Germiston virus	1	5	1
Ilesha virus	1	4	2
Lumbo virus	1	5	1
Sandfly Fever Naples virus	1	2	1
Ngari virus	7	4	2
Nyando virus	2	2	1
Oropouche virus	6	6	1
Pongola virus	8	6	2
Sandfly Fever Sicilian virus	1	2	2
Shuni virus	2	10	1
Tacaiuma virus	3	9	1
Tucunduba virus	2	2	1
Toscane virus	3	4	1
Witwatersrand virus	1	5	1

**TABLE 3:** “Consequence” sub-scores by agent.

Agent	Consequence sub-scores					
	Vulnerable populations	Status of immunity	Total # cases/Year globally	Rate of severe symptomatic cases	Case fatality rate	Long-term health impact
Yellow Fever virus	10	10	8	8	6	10
Dengue virus	10	10	10	8	2	10
Zika virus	10	10	8	4	2	8
Japanese encephalitis virus	10	10	8	10	10	10
West Nile virus	10	10	6	10	6	10
Bagaza virus	10	10	0	0	0	2
St. Louis encephalitis virus	10	10	4	10	8	10
Banzi virus	10	10	0	0	0	2
Ntaya virus	10	10	0	0	0	2
Murray Valley encephalitis virus	10	10	0	4	6	8
Spondweni virus	10	10	0	0	0	2
Uganda S virus	10	10	0	0	0	2
Usutu virus	10	10	0	6	0	4
Wesselsbron virus	10	10	0	2	0	2
Chikungunya virus	10	10	8	6	2	10
Western Equine encephalitis virus	10	10	2	6	6	10
Eastern Equine encephalitis virus	10	10	2	10	10	10
Venezuelan Equine encephalitis virus	10	10	2	6	2	8
Barmah Forest virus	10	10	2	2	0	4
Middelburg virus	10	10	0	2	0	2
Mayaro virus	10	10	0	0	0	2
Mucambo virus	10	10	0	0	0	2
Ndumu virus	10	10	0	0	0	2
O’Nyong-Nyong virus	10	10	0	2	0	4
Semliki Forest virus	10	10	0	0	0	4
Sindbis virus	10	10	2	2	0	4
Ross River virus	10	10	4	2	0	4
Orungo virus	10	10	0	0	0	2
Banna virus	10	10	0	2	0	4
Kamese virus	10	10	0	0	0	2
Mossuril virus	10	10	0	0	0	2
Vesicular stomatitis virus (Alagoas, Indiana, Jersey)	10	10	0	0	0	2
Chandipura virus	10	10	4	10	10	10
Rift Valley Fever Virus	10	10	8	6	10	10
La Crosse encephalitis virus	10	10	2	10	0	8
California encephalitis virus	10	10	0	4	2	8
Jamestown Canyon virus	10	10	2	10	2	8
Tahyna virus	10	10	2	0	0	2
Bunyamwera virus	10	10	0	0	0	2
Bwamba virus	10	10	0	4	10	6
Germiston virus	10	10	0	4	0	2
Ilesha virus	10	10	4	4	4	6
Lumbo virus	10	10	0	0	0	0
Sandfly Fever Naples virus	10	10	4	0	0	2
Ngari virus	10	10	2	6	10	8
Nyando virus	10	10	0	0	0	4
Oropouche virus	10	10	2	2	0	4
Pongola virus	10	10	0	0	0	2
Sandfly Fever Sicilian virus	10	10	2	0	0	2
Shuni virus	10	10	0	0	0	2
Tacaiuma virus	10	10	0	0	0	2
Tucunduba virus	10	10	0	2	0	6
Toscane virus	10	10	4	10	0	8
Witwatersrand virus	10	10	0	0	0	2

**TABLE 4:** “Mitigation” sub-scores by agent.

Agent	Mitigation sub-scores					
	Availability of rapid diagnostics	Availability of effective MCMs	Burden on public healthcare systems	Duration of home supportive care and time to RCV for most of the affected pop	Duration of hosp. For severely affected pop	Vector control measures
Yellow Fever virus	6	8	4	2	6	8
Dengue virus	4	8	6	2	10	8
Zika virus	4	10	4	2	6	8
Japanese encephalitis virus	6	8	6	2	10	8
West Nile virus	6	10	6	2	10	8
Bagaza virus	10	10	1	0	2	8
St. Louis encephalitis virus	6	10	6	2	10	8
Banzi virus	10	10	2	2	2	8
Ntaya virus	10	10	2	2	2	8
Murray Valley encephalitis virus	8	10	6	4	8	8
Spondweni virus	10	10	2	2	2	8
Uganda S virus	10	10	1	0	2	8
Usutu virus	8	10	4	4	4	8
Wesselsbron virus	10	10	3	2	4	8
Chikungunya virus	4	8	6	2	10	8
Western Equine encephalitis virus	8	10	6	4	8	8
Eastern Equine encephalitis virus	6	10	6	4	8	8
Venezuelan Equine encephalitis virus	8	10	6	4	8	8
Barmah Forest virus	8	10	3	4	2	8
Middelburg virus	10	10	2	2	2	8
Mayaro virus	8	10	2	2	2	8
Mucambo virus	10	10	2	2	2	8
Ndumu virus	10	10	2	2	2	8
O’Nyong-Nyong virus	10	10	3	2	4	8
Semliki Forest virus	10	10	3	2	4	8
Sindbis virus	8	10	2	2	2	8
Ross River virus	8	10	4	6	2	8
Orungo virus	10	10	3	2	4	8
Banna virus	10	10	3	2	4	8
Kamese virus	10	10	2	2	2	8
Mossuril virus	10	10	2	2	2	8
Vesicular stomatitis virus (Alagoas, Indiana, Jersey)	10	10	2	2	2	8
Chandipura virus	10	10	7	4	10	8
Rift Valley Fever Virus	8	10	6	2	10	8
La Crosse encephalitis virus	8	10	6	2	10	8
California encephalitis virus	8	10	5	2	8	8
Jamestown Canyon virus	8	10	6	2	10	8
Tahyna virus	10	10	2	2	2	8
Bunyamwera virus	10	10	2	2	2	8
Bwamba virus	10	10	4	2	6	8
Germiston virus	10	10	2	2	2	8
Ilesha virus	10	10	2	2	2	8
Lumbo virus	10	10	0	0	0	8
Sandfly Fever Naples virus	10	10	2	2	2	8
Ngari virus	10	10	4	2	6	8
Nyando virus	10	10	2	2	2	8
Oropouche virus	8	10	3	2	4	8
Pongola virus	10	10	2	2	2	8
Sandfly Fever Sicilian virus	10	10	2	2	2	8
Shuni virus	10	10	1	0	2	8
Tacaiuma virus	10	10	2	2	2	8
Tucunduba virus	10	10	5	2	8	8
Toscane virus	10	10	4	2	6	8
Witwatersrand virus	10	10	2	2	2	8

**TABLE 5:** Arbovirus risk assessment - criteria scoring definitions.

Transmission	
Vectors involved in transmission – The number and type of vectors that can contribute to the transmission of disease in human	
0 2 4 6 8 10	0–1 species of mosquitoes or flying insects 2–3 species of mosquitoes or flying insects 4–5 species of mosquitoes or flying insects 6–7 species of mosquitoes or flying insects 8–9 species of mosquitoes or flying insects 10 or more species of mosquitoes or flying insects
Reservoir/Host other than humans– The number of other potential host or carriers of the disease that can contribute to enhance transmission:	
0 2 4 6 8 10	None Only humans 1–2 host species 3–4 host species 5–6 host species 7 or most host species
Disease/Vector distribution – Number of countries impacted by the disease and vectors	
0 2 4 6 8 10	Less than 9 countries 10 to 24 countries 25 to 49 countries 50 to 74 countries 75 to 99 countries More than 100 countries

Consequence	
Vulnerable populations – The portion of the population susceptible to disease:	
0 2 4 6 8 10	None Small subset or group (e.g., rare genetic disorder) Only immunocompromised Immunocompromised and pregnant females Immunocompromised, pregnant females and children or elderly All
Status of immunity – The extent to which the global population may have immunity to the disease due to previous exposure or vaccination	
0 2 4 6 8 10	Close to 90–100% Significant (>60–90%) of population have immunity Moderate portion (30–60%) of population have immunity Previous vaccines or exposure may have reduced impact in about 10–30% Small subset (<10%) have immunity (e.g., from vaccination or previous exposure) No presumed immunity to disease in majority of the global population
Number of clinical cases per year – Number of individual infected per year	
0 2 4 6 8 10	Less than 100 100 –1K 1K–10K 10K – 100K 100K–1M 1M or higher
Rate of severe symptomatic cases – Individuals requiring hospitalization and supportive care	
0 2 4 6 8 10	Close to 0% 1 –9% 10–14% 15–19% 20–24% 25% or higher
Case fatality rate – The number of deaths from the disease per 100 diagnosed cases (or number of known cases):	
0 2 4 6 8 10	Close to 0% 1–9% 10–14% 15–19% 20–24% 25% or greater
Long–Term health effects – The extent to which a portion of the affected population may incur disability or require additional medical care beyond treatment for acute illness (sequelae):	
0 2 4 6 8 10	None and/or unknown due to too few cases Little to no long–term effects No functional effects, but follow–up medical care required Persists to a chronic stage for years and/or acute disease can reoccur Long–term functional impacts (e.g., flaccid paralysis, neurological) Some level of permanent incapacitation, requiring long–terms care (e.g., long term brain damage, liver damage)

Mitigation	
Availability of rapid diagnostics – The availability of diagnostics that can be rapidly deployed in response to a public health emergency:	
0 2 4 6 8 10	No need to deployed rapid diagnostics due to low morbidity and mortality Widely available and easy to deploy efficiently (e.g. lateral flow immunoassays) Available with some limitation to deploy widely (e.g. Real–Time PCR testing) due to lack of validation or availability of technology/reagents Available but difficult to deploy widely (e.g. Serological testing) due to lack of validation, expertise or availability of technology/reagents Available only at limited institutions (e.g., EUA required) or only approved outside the U.S. No Diagnostics currently available
Availability of effective medical countermeasures – The availability of efficacious medical treatments/countermeasures and extent to which they can be rapidly deployed in response to a public health emergency:	
0 2 4 6 8 10	No MCMs required or would be deployed or needed due to low morbidity mild illness Over the Counter MCM to treat or minimize the symptoms. Widely available and easy to deploy efficiently (e.g. Antiviral oral tablets/capsules) Widely available but difficult to deploy efficiently (e.g. intravenous–only drugs) or lacks efficacy Approved treatment or vaccine available in limited quantities No treatment beyond supportive care available
Burden on public health care systems – The potential burden to public health during and after an event, as measured by duration of medical countermeasure treatment and duration of hospitalization.	
Duration of home supportive care and time to recovery – The length of time for home supportive care for most of the affected population and the time to recovery:	
0 2 4 6 8 10	No symptoms 1–7 days 8–14 days 15–21 days 22–28 days >29 days
Illness severity – The type of illness associated with infection in severe cases:	
0 2 4 6 8 10	No illness or mild illness Fever (flu–like) Fever with other complications (paralysis, seizures, back pain, joint pain, myalgia etc.) Hemorrhagic Fever or Aseptic meningitis Neurological (encephalitis or meningitis) Neurological (encephalitis or meningitis) + Hemorrhagic Fever or other serious complications
Vector control to reduce disease persistence – The potential extent remediation efforts are effective to reduce vector persistence in the environment to reduce disease persistence in the population.	
0 2 4 6 8 10	No vector control measures required as disease is least likely to persist There are vector control measures that will eliminate disease persistence There are vector control measures that are highly effective There are vector control measures that are moderately effective There are vector control measures that have limited effectiveness There are no effective vector control measures

The scores for each agent were used to group arboviruses under consideration as high-risk, moderate-risk, or low-risk agents, as follows. One score had multiple components: “Duration of Home Supportive Care and Time to Recovery” and “Severity of Illness” (Table 5) were averaged to give a score for “Burden on Public Healthcare System,” as summarized in Figure 1.

Next, the resulting 13 factor scores, i.e., the composite scores noted above plus the remaining twelve single-criterion scores (Vector/s involved, Reservoir/Host other than humans, Disease/Vector distribution, Vulnerable population, Status of Immunity, Total number of cases/year globally, Rate of severe symptomatic cases, Case fatality rate, Long-term health impact, Availability of rapid diagnostics, Availability of effective medical countermeasure, Burden on public health and Vector control measures) for each arbovirus were analyzed in two ways: 1) a one-dimensional (1-D) ranking whereby the total sum and weighted sum (as defined in the next section) for each agent was tallied and the agents were ranked from lowest to highest; and 2) a two-dimensional (2-D) plot whereby the total sum and weighted sum of the sub-scores for the “transmission” (Vector/s involved, Reservoir/Host other than humans, and Disease/Vector distribution) branch of the hierarchy was plotted against the total and weighted sums of the sub-scores for the “consequences” (Vulnerable population, Status of immunity, Total number of cases/year globally, Rate of severe symptomatic cases, Case fatality rate, Long-term health impact) plus “mitigation” (Availability of rapid diagnostics, Availability of effective medical countermeasure, Burden on public health, Vector control measures) branches of the hierarchy (Figures 3–6).

### Criteria weighting

Weights were assigned to each criterion to account for factors that may carry more significance (i.e., public health impact) for the goals of arbovirus risk assessment. SMEs ranked each of the 13 criteria collectively, from one to three, where one described the least important criteria and three described the most important criteria. To demonstrate the MCDA methodology, two weighting schemes were tested: equal weighting (i.e., no weighting) and the weighting scheme derived from the SME’s inputs, as shown in Table 6. In the latter case, four criteria (Total number of cases/year globally, Rate of severe symptomatic cases, Case fatality rate, and Long term health impact) were given a 3x weight because of their high impact to public health, three criteria (Reservoir/Vertebrate host other than humans, Disease/Vector distribution, and Burden on public healthcare system) were given a 2x weight for their moderate impact to public health, and the last six criteria (Vector/s involved, Vulnerable populations, Status of immunity, Availability of rapid diagnostics, Available of effective medical countermeasures and Vector control measures to reduce disease persistence) were given a 1x weight for their lesser (or minimal) impact to public health. In addition to the above, weight assignments also took into consideration the following criteria: criteria that will not make a difference in the relative score for all the agents were assigned a lower weight (i.e., 1x); criteria that will have some impact on the different agents were assigned a moderate weight (i.e., 2x); and criteria that are critical to understanding the risk of agents with significant impact were assigned a higher weight (i.e., 3x).

**TABLE 6:** Criteria weighting schemes explored for Arbovirus Risk Assessment.

Criteria	Equal weighting	Proposed weighting scheme
(1) Vector/s involved	1x	1x
(2) Reservoir/Vertebrate Host other than Humans	1x	2x
(3) Disease/Vector Distribution	1x	2x
(1) Vulnerable Populations	1x	1x
(2) Status of Immunity	1x	1x
(3) Total Number of Cases/year globally	1x	3x
(4) Rate of Severe Symptomatic Cases	1x	3x
(5) Case Fatality Rate	1x	3x
(6) Long-Term Health Impact	1x	3x
(1) Availability of Rapid Diagnostics	1x	1x
(2) Availability of Effective Medical Countermeasures	1x	1x
(3) Burden on Public Healthcare System	1x	2x
(4) Vector Control Measures to reduce Disease Persistence	1x	1x

For both cases, criteria and weights were combined into a single score (A) by summing all the weighted numerical values (a_ij_,w_j_), where a_ij_ represents a criteria score and w_j_ is the criteria weighting value:

$$A=\sum_{j=1}^{n}a_{ij}\;\cdot \;w_{j}$$

To enable comparison of results using different weighting values, normalized scores were used, whereby the total or sub-total scores were normalized to those of a hypothetical agent that received 10s for all 13 criteria scores.

### Agent information

Critical data were captured from published literature and relevant databases for scoring pathogens against the 13 criteria noted above, for 54 arboviruses (Table 1). Fourteen Flaviviridae, 13 Togaviridae, 2 Sedoreoviridae, 4 Rhabdoviridae and 21 Peribunyaviridae, Nairoviridae, or Phenuiviridae were included in the analysis based on relevance for the risk assessment. Heartland virus, severe fever thrombocytopenia syndrome virus, Crimean Congo Hemorrhagic Fever virus, Omsk Hemorrhagic Fever virus, Kyasanur Forest Disease virus, Tick-borne encephalitis virus, Powassan virus, Bourbon virus, Colorado tick fever virus; other arboviruses transmitted by fleas or ticks; and arboviruses transmitted by mosquito, sandflies, and midges that are not known to cause disease in humans (e.g., Getah virus, Potosi virus) were not included in the risk assessment.

Development of the agent fact sheet used peer-reviewed open literature, such as Medline, PubMed, Google Scholar, CDC ArboNET or Arbovirus Catalog (CDC, 2024c), and other unclassified data sources, and was followed by extensive review by SMEs. In all cases, SME judgement was relied upon to provide concurrence on the best available data or basis for scoring. SMEs reviewed the data provided in the fact sheet for accuracy and relevance, as well as the scores assigned to each data category. Comments received from SMEs were verified through literature search and review of unpublished data and incorporated into the agent fact sheet, with scoring adjusted as necessary.

### Decision support framework (DSF)

**FIGURE 2:**
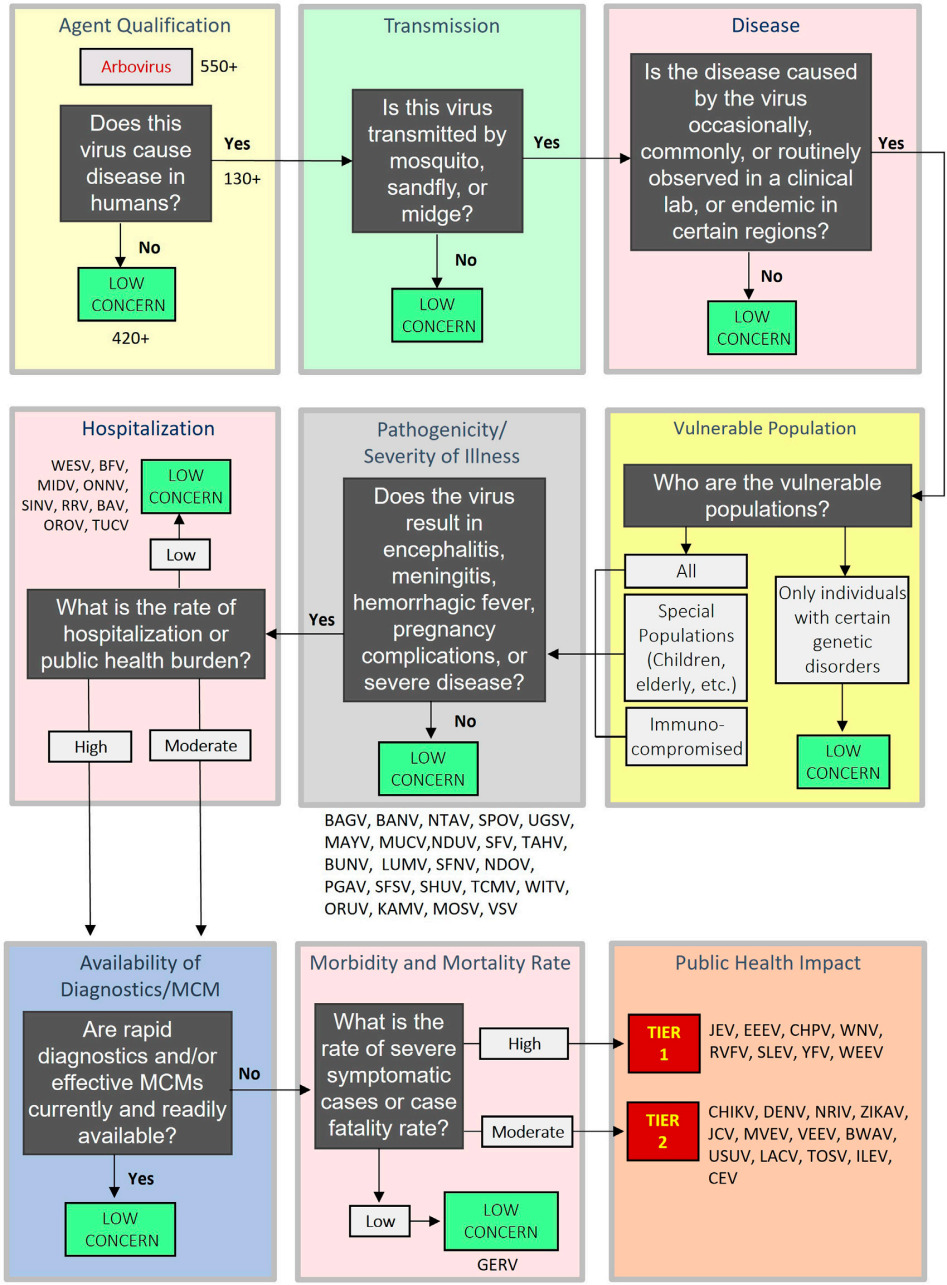
Schematic of the Decision Support Framework logic tree showing assignment for Arbovirus Risk Assessment.

The DSF approach applies key criteria using a logic tree format to identify pathogens that may be of sufficiently low concern that they can be ruled out from further consideration as a Tier 1 (high) or Tier 2 (moderate) risk for a public health impact and are Tier 3 (low risk), as described previously (Pillai S. P. et al., 2022; Pillai S. P. et al., 2022; Pillai et al., 2023a; Pillai et al., 2023b). The DSF is complementary to the MCDA approach and avoids the possible unintended numerical equivalences that may occur using weighted, or unweighted sums (Pillai S. P. et al., 2022; Pillai S. P. et al., 2022; Pillai et al., 2023a; Pillai et al., 2023b). Additionally, the DSF considers the potential impact associated with arbovirus vs. the public health risk. Those arboviruses that exceed all criteria thresholds are considered potential Tier 1 (high-risk) or Tier 2 (moderate-risk) for public health. Criteria include Agent Qualification, Transmission, Disease, Vulnerable Population, Pathogenicity/Severity of Illness, Hospitalization, Availability of Diagnostics/Medical Countermeasures and Morbidity and Mortality (Figure 2). SME judgment based on data captured in the agent fact sheets provide the basis for scoring. In general, criteria which received a score of zero, two, or four in some cases typically served as a basis for a “low concern” qualitative assessment. In contrast to the MCDA approach, which uses a graded scoring system for ranking agents, the DSF approach can rule out an arbovirus for risk consideration using a single (low scoring) criterion. Many of the criteria overlap between the MCDA and DSF approaches.

## Results

### Data gaps and quality

When considering 54 arboviruses across a broad range of attributes, data gaps and variability in data quality are inevitable. Data availability in the open literature tends to parallel scientific inquiry for the virus; for example, Disease/Vector distribution is challenging, simply because a vector isolated as part of surveillance and carrying the virus does not mean that it will or even could infect humans or another host. Regarding the factor of Vulnerable Population, sometimes children and the elderly tend to be more susceptible than adults but nevertheless, all are susceptible to the disease. The number of cases per year globally is often challenging to predict because of underreporting. In addition, due to the underreporting or lack of appropriate diagnostics in some low-income countries, the data for Rate of Severe Symptomatic Cases and Case Fatality, Mortality, or Morbidity rates associated with the disease may be skewed.

### Unweighted rankings

**FIGURE 3:**
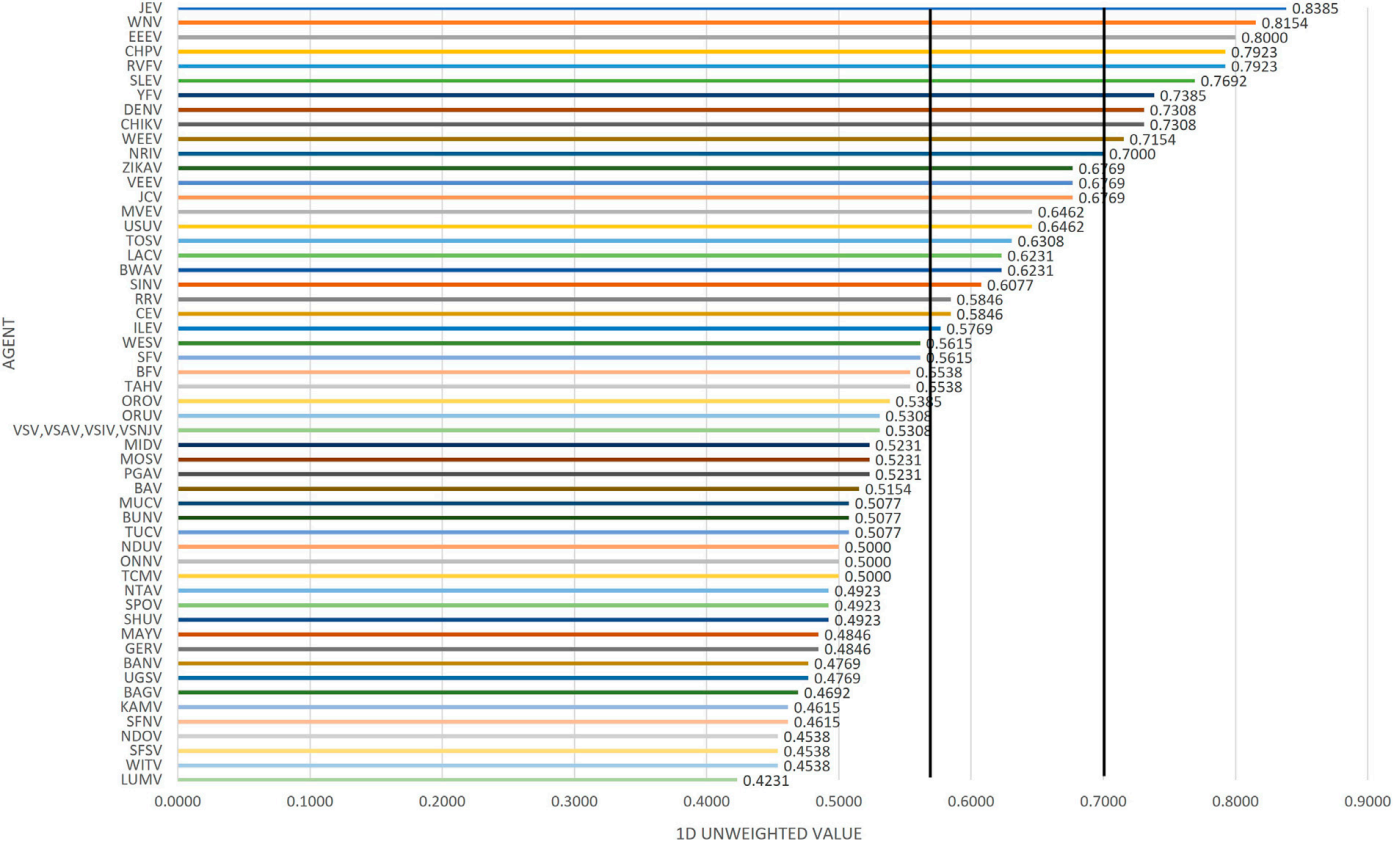
1D plot of unweighted scoring results for Arbovirus Risk tiering.

**FIGURE 4:**
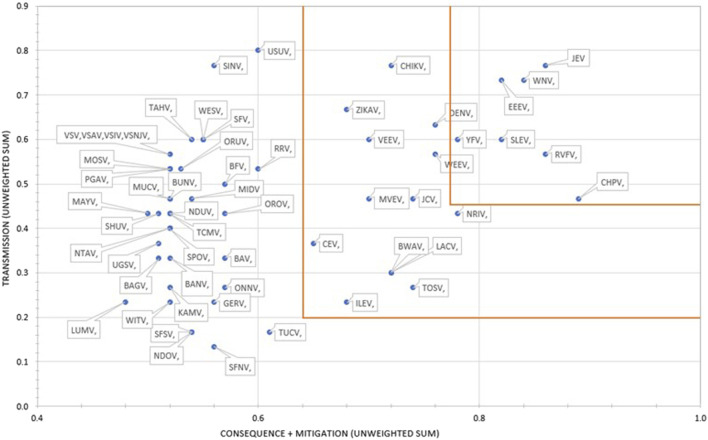
2D plot of unweighted scoring results for Arbovirus Risk tiering.

Initial review of the 1D results, whereby the total scores for all 54 arboviruses are compared (Figure 3), generally indicated minimal difference in the high-risk agents (Tier 1) identified at the top of the rank-ordered list when compared with 2D plots. Similarly, for the 2D plots, whereby summated sub-scores for transmission and consequence + mitigation for all 54 agents were plotted against each other (Figure 4), Arboviruses generally found in the upper right quadrant of the plot mostly fall in similar placement in the 1D plots; however, there were some minor exceptions. Analysis of equally weighted scores for both the 1D and 2D plots indicated that there were general trends in the data, and the results were somewhat consistent with minor differences (see Figures 3, 4). For example, in the 1D plot, if the threshold was designated as 0.70 for Tier 1 risk and 0.57 for Tier 2 risk (Figure 3); and in the 2D plot, if the thresholds for the x-axis and y-axis scores for high-risk arboviruses were designated as 0.77 and 0.44 for Tier 1 risk and 0.64 and 0.20 for Tier 2 risk, respectively, then this led to the notional thresholds for risk categorization as shown in Figure 4.

### Weighted rankings

**FIGURE 5:**
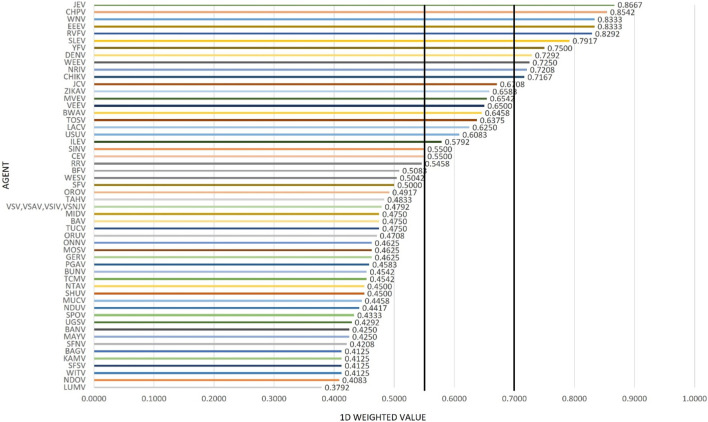
1D results for the proposed weighting scheme for Arbovirus Risk tiering.

**FIGURE 6:**
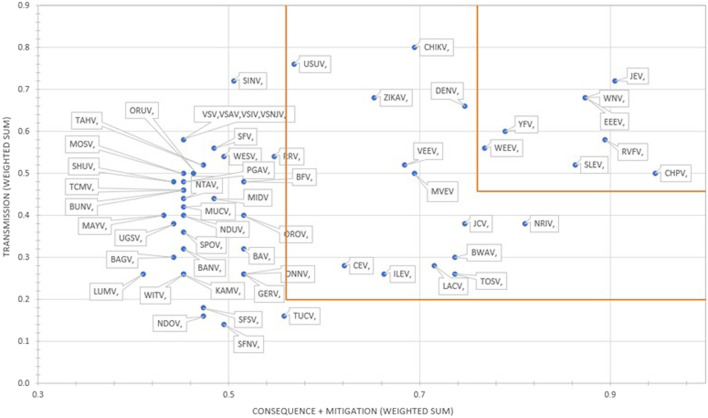
2D results for the proposed weighting scheme for Arbovirus Risk tiering.

The results using the proposed weighting scheme described in Table 6 for 1D and 2D formats are shown in Figures 5, 6, respectively. As observed with the unweighted data, the general trend in the data was consistent with the weighted data with minor variations; however, any designation of a minimal score as a basis for public health risk—whether the total score in the 1D plot, or sub-scores corresponding to x- and y-axes values in the 2D plots—resulted in Tier 1 or Tier 2 category with some minor differences. For example, in the 2D plot, if we designated the lowest x-axis and y-axis scores allowed for classification as a Tier 1 risk agent to be 0.76 and 0.45, respectively, and for Tier 2 risk agents to be 0.56 and 0.20, based on SME input, as illustrated in Figure 6, we found JEV, EEEV, CHPV, WNV, RVFV, SLEV, YFV and WEEV fell into the Tier 1 risk category, whereas CHIKV, DENV, NRIV, ZIKAV, JCV, MVEV, VEEV, BWAV, USUV, LACV, TOSV, ILEV, and CEV fell into the Tier 2 risk category.

### Decision support framework

To evaluate the 54 arboviruses using the DSF approach, we leveraged the agent fact sheets developed for this analysis. As shown in Figure 2, the DSF is a logic tree with a series of key categorical questions that can be used to guide the risk assessment for each arbovirus, ultimately informing the user of its tiered impact on public health. The questions were placed into eight general categories within the logic tree that comprises the DSF: 1) Agent Qualification; 2) Transmission; 3) Disease; 4) Vulnerable Population; 5) Pathogenicity/Severity of Illness; 6) Hospitalization; 7) Availability of Diagnostics/MCM; and 8) Morbidity and Mortality Rate. Starting with agent qualification, the user answers the series of questions following the logic tree for each arbovirus, with the result that arbovirus (es) of low concern would be potentially eliminated at each category of the tree or continue through the remainder of the tree as a moderate-to high-concern agent. If an agent was identified as low-concern, it could be excluded for consideration as a Tier 1 or Tier 2 agent (high or moderate public health risk, respectively). If an agent exceeded all low-concern criteria thresholds across the DSF, it was categorized as either Tier 1 or Tier 2. All 54 arboviruses analyzed using the DSF were not found to be of low concern for the first four categories. The “Pathogenicity/Severity of Illness” category decision eliminated 23 arboviruses that were deemed to be of low concern: BAGV, BANV, NTAV, SPOV, UGSV, MAYV, MUCV, NDUV, SFV, TAHV, BUNV, LUMV, SFNV, NDOV, PGAV, SFSV, SHUV, TCMV, WITV, ORUV, KAMV, MOSV, and VSV (see Figure 2 for a visual representation of the results). The next category, “Hospitalization,” eliminated nine additional low-concern agents: WESV, BFV, MIDV, ONNV, SINV, RRV, BAV, OROV, and TUCV. For “Diagnostics/MCM,” no low-concern agents were identified. For “Morbidity and Mortality Rate,” one agent was identified as low-concern: GERV. The 13 arboviruses identified as having a moderate rate of severe symptomatic cases or case fatality rate were categorized as Tier 2 (moderate-risk) agents: CHIKV, DENV, NRIV, ZIKAV, JCV, MVEV, VEEV, BWAV, USUV, LACV, TOSV, ILEV, and CEV, and the eight arboviruses having high rates of such cases were categorized as Tier 1 (high-risk) agents: JEV, EEEV, CHPV, WNV, RVFV, SLEV, YFV, WEEV.

## Discussion

Arboviruses are a diverse group of viruses that pose a substantial threat to human and animal health. With increasing globalization and climate change, the geographical range of arboviruses and their transmitting vectors is expanding, leading to the emergence, re-emergence, and wider transmission and distribution of arboviral diseases globally. Understanding the vector biology, pathogenesis, transmission, and impact to public health is crucial for the implementation of effective surveillance, prevention, and vector control efforts; as well as for strategic investment, planning, preparedness, and response-related efforts.

### Disease transmission

Arboviruses are primarily transmitted through the bites of infected arthropod vectors, such as mosquitoes, sandflies, midges, and ticks. The transmission cycle involves the virus circulating between vertebrate hosts, such as birds, humans, and other mammals, and the arthropod vectors. Factors influencing transmission dynamics include vector type, population, distribution, and abundance; viral replication rates and circulation in the blood; host susceptibility; and environmental conditions.

### Surveillance and detection

Effective control measures are guided by comprehensive surveillance and monitoring of mosquito and tick populations and disease emergence, re-emergence, or prevalence in hosts (e.g., birds, other animals). Surveillance systems provide data on vector type, distribution, abundance, and infection rates, enabling targeted interventions for disease control and prevention and early detection of emerging threats for effective mitigation. Techniques such as mosquito trap monitoring, larval surveys, pathogen detection, and disease surveillance or public health surveillance are essential components of surveillance efforts to provide early warning for public health intervention.

### Vector control

Effective and integrated vector control measures play a crucial role in mitigating the transmission of arboviruses and reducing the burden of associated diseases. Targeting mosquitos and other vectors in their breeding habitats is a key strategy for reducing vector populations and interrupting the transmission cycle of arboviruses. Mosquito larval and adult control measures include habitat modification; source reduction; biological control using natural predators or pathogens; use of Mosquito Bits, bug zappers, and mosquito traps; environmental monitoring and removal of sources for breeding and spraying for vector control measures; and the use of chemical larvicides. Integrated vector management approaches that combine multiple control methods are often the most effective in controlling vector populations (Côrtes et al., 2023).

Community engagement and participation are essential for vector control measures to be successful. Public education, awareness campaigns, and community-based interventions empower individuals to take proactive measures to reduce mosquito breeding sites for vector control measures, remove any source for breeding (e.g., standing water), protect themselves from bites (e.g., by using N, N-diethyl-meta-toluamide [DEET], mosquito nets), and understand the clinical symptoms to seek timely medical care. Community partnerships could also facilitate the implementation of sustainable and effective vector control strategies tailored to local contexts (Soria et al., 2024)

Advances in technology offer new opportunities for vector control measures, including genetically modified mosquitoes, sterile insect technique (SIT) (Wang et al., 2021), and Wolbachia-based interventions (Branda et al., 2004; Montenegro et al., 2024). These innovative approaches have the potential to complement traditional vector control and further contribute to its effectiveness; and reduce reliance on chemical insecticides or other physical methods. However, ethical, regulatory, and social considerations must be carefully addressed to ensure responsible deployment and acceptance of new technologies.

### Public health preparedness and response

To support disease surveillance, management and clinical intervention, it will be good to consider the need for supportive care, ventilators, and effective over-the-counter medications to address symptoms and provide relief; development of effective vaccines for arbovirus-related diseases that currently lack an effective vaccine; development of effective antivirals; and the development of rapid diagnostics such as lateral flow immunoassays (LFIA), enzyme-linked immunosorbent assays (ELISA) for serology and antigen detection, and real-time polymerase chain reaction (RT-PCR).

To support these proactive approaches, pathogen selection and prioritization for a specific intended use could be carried out using a formalized risk ranking process with selected weighted criteria to meet a required objective (McFadden et al., 2016). Similar processes have been previously used in both public health and veterinary health spheres (McFadden et al., 2016; Cardoen et al., 2009; Havelaar et al., 2010; Ciliberti et al., 2015; Roelandt et al., 2017) to support prevention, early warning surveillance and control measures for disease incursion. Although there is no universal methodology for risk ranking, it is important that risk ranking exercises use a structured approach, which is transparent and consistently documented to be reproducible. MCDA- and DSF-based risk assessments are already recognized as useful tools to support select agent and toxin designations (Pillai S. P. et al., 2022; Pillai S. P. et al., 2022; Pillai et al., 2023a; Pillai et al., 2023b)

Here we investigated using MCDA and DSF as a structured approach to inform the risk associated with arboviruses to support public health investments, planning, preparedness, and response efforts. The approach was flexible with the ability to adjust both the criteria and their weighting based on SME input and contribution.

MCDA (unweighted and weighted) and DSF methods were chosen for their individual merits and to provide confirmation of the observed results. While both methods enabled a risk-informed comparison of a diverse set of pathogens in a structured way, the MCDA results were challenged by a continuum of scores that did not suggest natural thresholds for classification of high-risk vs. moderate risk vs. low risk. Alternatively, the DSF employs a series of criteria thresholds to identify pathogens for consideration as high-risk vs. moderate-risk vs. low-risk and provides clear classification assignments.

The finding that both approaches arrived at a consistent set of pathogens for consideration as high-risk vs. moderate-risk vs. low-risk (based on threshold settings) further supports their usefulness for funding/investment decisions as well as for planning, preparedness, and response to arbovirus-related public health efforts. These methodologies can also be leveraged to evaluate new and novel arboviruses that may emerge to gain a better understanding of their potential risk and impact to public health.

Application of the methodology across a large and diverse pathogen set, while helping to demonstrate the robustness of the approach, highlighted the challenge of how to handle data gaps for many pathogens. At times, the uncertainties in published data for some criteria required SME review of the data and discussions on how to account for the uncertainties in the data. Although there are still some data gaps in understanding disease severity and illness, number of cases globally, mortality rates, etc. for some agents, it should be noted that these risk assessment approaches are meant to evolve as new data becomes available, from future research or outbreaks. The MCDA and DSF represent a data driven approach for Arbovirus risk assessment to support funding decisions, prioritization, planning, preparedness, and response efforts.

There is no single definitive “global prioritization list” for vector-borne diseases, but the World Health Organization (WHO) and other health organizations identify diseases posing significant threats based on factors such as impact, potential for spread, and existing tools for prevention and control; key priority vector-borne diseases often include malaria, dengue, yellow fever, Lyme disease, Zika, chikungunya fever, Japanese encephalitis, Oropouche fever, and West Nile virus (WHO. Vector-borne diseases, 2025).

However, in the case of the authors’ analysis herein, the MCDA results were compared to laboratory safety recommendations and the assignment of these arboviruses to BSL-2 or -3 based, in part, on risk assessments derived from information provided by a worldwide survey of laboratories working with arboviruses, newly published reports on the viruses, reports of laboratory infections, and discussions with scientists working with each virus (CDC/NIH, 2020). Of the 8 arboviruses considered high risk by MCDA (Figure 6), 5 (62.5%) had a recommendation of BSL-3 (JEV, EEEV, RVFV, YFV, WEEV). Of the 13 arboviruses considered moderate risk by MCDA, 3 (23.1%) were BSL-3 agents (VEEV, CHIKV, NRIV) and of the 34 arboviruses considered low risk by MCDA, 4 (11.8%) were BSL-3 agents (WESV, SFV, BAV, GERV). Thus, while there is an association between arbovirus risk tiering by MCDA and biosafety laboratory risk, there are additional factors that are important in assessing public health risk associated with natural events.

Several arboviruses have been determined to have the potential to pose a severe threat to both human and animal health and have been classified as Select Agents to support bioterrorism prevention, preparedness, and response. These viruses are EEEV, RVFV, VEEV, and ASFV (CDC/USDA, 2025). The agents on the Select Agent list, as well as new agents, are reviewed on a biennial basis to determine whether they should be added to the list, remain, or be removed. A MCDA method was developed to facilitate this review (Pillai S. P. et al., 2022; Pillai S. P. et al., 2022; Pillai et al., 2023a; Pillai et al., 2023b).

## Conclusion

Arboviruses represent a significant public health challenge, with their transmission dynamics influenced by a complex interplay of ecological, environmental, and socio-economic factors. Effective planning, preparedness, surveillance, prevention, and response strategies are essential for mitigating the impact of arboviral diseases on global public health. Continued research efforts to better understand the vector biology and disease pathogenesis of arboviruses, and intervention and mitigation are crucial for developing innovative interventions to combat these emerging threats.

Vector control measures are essential for preventing arbovirus-related diseases and safeguarding public health. By integrating vector surveillance, disease surveillance, vector control measures, community engagement, leveraging emerging and advanced technologies, and public health preparedness efforts, countries can develop comprehensive strategies to combat arboviral transmission and mitigate disease. Continued research, collaboration, and innovation are crucial for identifying and addressing challenges and adapting vector control efforts to evolving threats posed by arboviruses.

## Data Availability

The raw data supporting the conclusions of this article will be made available by the authors, without undue reservation.
